# Subjective sleep quality and association with depression syndrome, chronic diseases and health-related physical fitness in the middle-aged and elderly

**DOI:** 10.1186/s12889-021-10206-z

**Published:** 2021-01-19

**Authors:** Min-Fang Hsu, Kang-Yun Lee, Tsung-Ching Lin, Wen-Te Liu, Shu-Chuan Ho

**Affiliations:** 1 Department of Nursing, Yuanpei University of Medical Technology, Hsinchu City, Taiwan; 2 Division of Pulmonary Medicine, Department of Internal Medicine, Shuang Ho Hospital, Taipei Medical University, New Taipei City, Taiwan; 3 Division of Pulmonary Medicine, Department of Internal Medicine, School of Medicine, College of Medicine, Taipei Medical University, Taipei City, Taiwan; 4 Department of Physical Medicine and Rehabilitation, Far Eastern Memorial Hospital, New Taipei City, Taiwan; 5 Shih-Chien Rehabilitation Clinic, Medical Deputy Superintendent, Taipei City, Taiwan; 6 Division of Geriatric Medicine, Department of Family Medicine, Taipei Medical University, 250 Wuxing Street, Taipei City, Taiwan; 7 School of Respiratory Therapy, College of Medicine, Taipei Medical University, Taipei City, Taiwan; 8 School of Respiratory Therapy, College of Medicine, Taipei Medical University, 250 Wuxing Street, Taipei City, 11031 Taiwan

**Keywords:** Subjective sleep quality, Pittsburgh sleep quality index, Health-related physical fitness, Depression symptoms, Arthritis

## Abstract

**Background:**

As a complex phenomenon, sleep quality is difficult to objectively define and measure, and multiple factors related to sleep quality, such as age, lifestyle, physical activity, and physical fitness, feature prominently in older adult populations. The aim of the present study was to evaluate subjective sleep quality using the Pittsburgh Sleep Quality Index (PSQI) and to associate sleep quality with health-related physical fitness factors, depressive symptoms, and the number of chronic diseases in the middle-aged and elderly.

**Methods:**

We enrolled a total of 283 middle-aged and elderly participants from a rehabilitation clinic or health examination department. The PSQI was used to evaluate sleep quality. The health-related fitness assessment included anthropometric and physical fitness parameters. Depressive symptoms were measured with the Center for Epidemiologic Studies Depression Scale (CES-D) short form. Data were analyzed with SPSS 18.0, and descriptive statistics and logistic regression analysis were used for the analyses.

**Results:**

Overall, 27.9% of participants in this study demonstrated bad sleepers (with a PSQI score of > 5), 10.2% of study participants frequently used sleep medication to help them fall asleep, and 6.0% reported having significant depressive symptoms (with a CES-D score of ≥10). There are two major findings: (1) depression symptoms, the number of chronic diseases, self-rated health, and arthritis were significantly associated with a poor sleep quality, and (2) the 2-min step test was associated with longer sleep latency. These results confirmed that the 2-min step was associated with a longer sleep latency among the health-related physical fitness items.

**Conclusions:**

Our study found that depressive syndrome, chronic disease numbers, a poor self-rated health status, and arthritis were the main risk factors that influenced subjective sleep quality.

## Background

As a complex phenomenon, sleep quality is difficult to define and measure objectively [1], and it is not directly associated with sleep quantity. The best assessment of sleep quality includes both subjective and objective measures [2]. Polysomnography (PSG) is the gold standard objective measure of sleep [3], and the Pittsburgh Sleep Quality Index (PSQI) is a widely used self-administered sleep questionnaire [1]. Self-reported sleep does not correlate well with PSG-defined sleep [4]. Multiple factors are related to sleep quality in old age, including lifestyle, physical activity, and physical fitness; moreover, alcohol consumption patterns [5], depressive symptoms [6], and rheumatoid arthritis (RA) [7] may also affect sleep quality. Among multiple factors related to sleep quality, recent studies began to focus on physical activity and physical fitness [8, 9].

Sleep is important to physical, cognitive, and psychological health. Sleep quality changes as a function of normal aging, there is no association between subjective sleep quality and cognitive performance in healthy young adults [10], and only half of middle-aged and elderly Chinese reported good sleep quality [11]. Age-related sleep changes may lead to poor sleep quality in older adults with physical or psychiatric disorders [12]. Many middle-aged and older adults do not sleep well, and they are more likely to take pharmacological sleep aids [13]. Studies revealed that 49% of community-dwelling older adults reported poor sleep quality [6], and 41.9% of adults aged 60 years or older suffer from sleep disturbances in Taiwan [14]. Sleep disturbances are also associated with lower physical fitness levels, a reduced health-related quality of life [15], nocturia, poor self-reported functional status, and one’s mental status [14]. Moreover, symptoms of depression were identified as factors most significantly associated with poor sleep quality among older adults [6, 14].

Young female adults with poor sleep quality are more likely to have lower levels of physical activity (muscle endurance, flexibility, and cardiovascular fitness) [16]. Studies revealed the benefits of physical activity and regular exercise in improving sleep quality and reducing the occurrence of sleep disturbances [8, 9, 17] in middle-aged and elderly adults. Physical activity in middle-aged and elderly adults differs from that of younger populations. Health-related fitness is the ability to become and remain physically healthy. Health-related physical fitness includes muscle strength, muscle endurance, cardiovascular endurance, joint flexibility, and body composition (American Alliance for Health, Physical Education, Recreation and Dance, 1980). Few studies have investigated relationships of depression with the quality of sleep and health-related physical fitness level. Therefore, the aim of the present study was to evaluate the subjective sleep quality using the PSQI, depression, and chronic diseases and their associations with the influence of health-related physical fitness factors in community-dwelling middle-aged and elderly.

## Methods

### Design and sample

A cross-sectional survey was conducted in our study. We recruited older adults in northern Taiwan from August 2010 to July 2013. The inclusion criteria of the sample were as follows: (a) aged 40 years or older; (b) with no ambulatory problems or using any assistance for walking; and (c) able to communicate verbally. Those who had severe cardiovascular disease, neurological disease, or musculoskeletal impairment were excluded. In total, the sample size was 283 individuals. The study was approved (no. 098101–3) by the Ethics Committee of Far Eastern Memorial Hospital, and all subjects provided written informed consent before they were allowed to participate in the study.

### Measures

The evaluated characteristics of respondents included sex, age, marital status, educational level, religious beliefs, number of current diseases (hypertension, heart disease, diabetes mellitus, pulmonary disease, peptic ulcers, liver disease, arthritis, osteoporosis, and cancer), smoking and alcohol consumption habits, tea and coffee consumption, and regular exercise (more than 30 min at least 3 days per week) [6]. Self-ratings of the health status, which reflected an individual’s personal perception of their overall health condition, were indicated on a 5-point Likert scale (scored 1 = very poor to 5 = excellent).

The PSQI was used to evaluate sleep quality [1], and the Chinese version (CPSQI) was used in this study [18]. The CPSQI had an overall reliability coefficient of 0.82 ~ 0.83 for community-dwelling adults with primary insomnia and control subjects, and also acceptable test-retest reliability over a 14 ~ 21-day interval with a coefficient of 0.85 for all subjects and 0.77 for primary insomniacs [18]. Participants self-rate their sleep situation with respect to seven components: sleep duration, sleep disturbance, sleep latency, daytime dysfunction, sleep efficiency, subjective sleep quality, and use of sleep medication in the past month. The score of each component ranges 0 ~ 3, and the sum of these component scores, ranging 0 ~ 21, is the total score that serves as a measure of sleep quality. A global score higher than five was defined as an indicator of bad sleeper [1]. In our study, we also used the original scores of the seven components and individually recoded them as dichotomous variables to examine the association of the correlates with these seven components.

The health-related fitness assessment included the following anthropometric and physical fitness measurements: (1) body weight and height were measured with a calibrated scale (within ±0.1 cm), and participants were asked to wear light clothes and no shoes. Body-mass index (BMI) calculated according to the body weight (kg) divided by the height (in m^2^); (2) mid-arm circumference, waist circumference, and buttock circumference measured according to standard methods; (3) body composition assessed using a bioelectrical impedance analysis (TANITA’s body composition analyzer, TBF 300, Tokyo, Japan) to derive the percentage of body fat, fat mass, and fat-free mass; and (4) physical activity measured using the International Physical Activity Questionnaire (IPAQ) short version, which comprises four generic items. The questionnaire was developed in Geneva in 1998 and has undergone extensive reliability and validity testing across many countries and has been translated into several different languages. We adopted the Taiwanese version [19]. Participants were asked to report their physical activity during the previous 7 days as an estimate of their habitual level of physical activity. IPAQ scores were categorized into three levels of physical activity: low, moderate, and high.

Physical fitness assessment: We used a set of senior fitness test tools developed by Accuratus International Health Company of Taiwan [20]. The test contained the following seven items: (1) sit-and-reach test, which is commonly used to measure the flexibility of the lower back; (2) one-leg stance test, which is used to assess static balance; (3) 2-min step test, which assesses cardiovascular endurance; (4) back muscle strength, which measures the maximal isometric strength of the trunk muscles in a standing posture with 30° lumbar flexion using a back muscle strength meter; (5) 30-s chair-stand test, which is used to assess lower-body strength; (6) grip strength, which measures the muscle strength of the upper arms; and (7) up-and-go test, which measures the speed, agility, and balance when the body is moving. For grip strength (no. 6), participants were asked to stand and grasp a grip strength-measuring device (Jamar® Plus+ Digital Hand Dynamometer; Sammons Preston, Rolyon, Bolingbrook, IL, USA). They performed this action three times, and the best score was recorded. All physical fitness measurements were performed by well-trained personnel, and the results were recorded on a computer.

Depressive symptoms were measured using the Center for Epidemiologic Studies Depression Scale (CES-D) short form [21]. The Chinese version of 10 self-reported items was used to measure depressive symptoms that occurred in the previous week. The score of each question ranges 0 ~ 3, with the total score ranging 0 ~ 30; we considered that a score of ≥10 indicated depression [21].

### Statistical analysis

Descriptive statistics were recorded for each variable of the characteristics of subjects and health-related fitness, with quantitative data shown as the mean and standard deviation (SD). Differences in comparisons of two group were analyzed with an independent *t*-test, Chi-squared test, Fisher’s exact test, and Mann-Whitney U-test. Spearman’s correlation was used for the PSQI and health-related fitness. A logistic regression analysis was performed to assess the characteristics and physical fitness associated with PQSI variables. The level of significance was set to α = 0.05. Data were analyzed with SPSS for Windows vers. Ninteen software (IBM, SPSS, Chicago, IL, USA).

## Results

In total, 283 individuals completed the entire physical fitness evaluation, and the PSQI questionnaire. Table 1 lists participants’ characteristics. The mean age was 59 ± 7.3 years, and approximately third-fourths of the study population were female and had religious beliefs. Only 5.3% of participants had a smoking history, 18% used alcohol at least once a week, about 60% used tea and coffee at least once a week, 49.5% engaged in regular exercise, only 5.7% did not achieve a moderate to high level of physical activity, 57.2% (162 subjects) had chronic disease, and 6% had depressive symptoms. Older adults, those with less alcohol use, and those with fewer depression symptom had significantly better sleep. A greater number of chronic diseases, especially heart disease and arthritis, were associated with poor sleep quality.

**Table 1:** Characteristics of the study subjects (*N* = 283)

		Quality of sleep		*p* value
Variable	*n* (%)	Good *n* (%)	Poor *n* (%)	
**Age (years) (59 ± 7.3)**				**0.020**^**#**^
< 65	227 (80.2)	161 (70.9)	66 (29.1)	
65 ~ 74	45 (15.9)	38 (54.4)	7 (15.6)	
≥ 75	11 (3.9)	5 (45.5)	6 (54.5)	
Sex				0.317
Male	60 (21.2)	47 (78.3)	13 (21.7)	
Female	223 (78.8)	157 (71.2)	66 (22.6)	
Religious beliefs				0.513
No	61 (21.6)	46 (75.4)	15 (24.6)	
sYes	222 (78.4)	158 (71.2)	64 (28.8)	
Smoking history				0.284
No	268 (94.7)	195 (72.8)	73 (27.2)	
Yes	15 (5.3)	9 (60.0)	6 (40.0)	
**Alcohol consumption**				**0.048**
No	232 (82.0)	162 (69.8)	70 (30.2)	
At least once a week	51 (18.0)	42 (82.4)	9 (17.6)	
Tea consumption				0.407
No	116 (41.0)	85 (73.3)	31 (26.7)	
At least once a week	167 (59.0)	119 (72.3)	48 (28.7)	
Coffee consumption				0.521
No	110 (38.9)	79 (71.8)	31 (28.2)	
At least once a week	173 (61.1)	125 (72.3)	48 (27.7)	
Regular exercise				0.526
No	143 (50.5)	101 (70.6)	42 (29.4)	
Yes	140 (49.5)	103 (73.6)	37 (26.4)	
**Chronic disease** ^**a**^				**0.025**
No	121 (42.8)	95 (78.5)	26 (21.5)	
Yes	162 (57.2)	109 (67.3)	53 (32.7)	
**Heart disease**				**0.017**
No	250 (88.3)	186 (74.4)	64 (25.6)	
Yes	33 (11.7)	18 (54.5)	15 (45.5)	
**Arthritis**				**0.002**
No	258 (91.2)	193 (74.8)	65 (25.2)	
Yes	25 (8.8)	11 (44.0)	14 (56.0)	
Physical activity ^b^ (Mets)				0.949^#^
Low	15 (5.7)	11 (73.3)	4 (26.7)	
Moderate	135 (51.1)	95 (70.4)	40 (29.6)	
High	114 (43.2)	83 (72.8)	31 (27.2)	
**Depressive symptoms** ^**c**^				**0.004**
No	264 (94.0)	195 (73.9)	69 (26.1)	
Yes	17 (6.0)	7 (41.2)	10 (58.8)	

^a^ Chronic diseases included heart disease, arthritis, hypertension, diabetes mellitus, lung disease, peptic ulcers, liver disease, osteoporosis, gout, and cataracts. Heart disease and arthritis were significant, while the others were not. ^b^ mean ± standard deviation: 4129.72 ± 4811.94; ^c^ CES-D score:2.74 ± 4.02. ^#^ Fisher’s exact test. Mets, metabolic equivalents

Overall, Table 2 shows the distribution of the quality of sleep according to the PSQI questionnaire. Global scores indicated that 27.9% of participants had poor sleep quality. The majority of participants (69.6%) reported that they usually had more than 7 h of sleep at night, most (86.9%) reported only a slight problem with sleep disturbances, and 26.9% of participants reported needing more than 30 min to fall asleep. Results of sleep efficiency revealed that three-quarters of participants had sleep efficiency exceeding 87.6%; however, approximately one-third of participants self-rated their sleep quality as poor, and most (83.7%) had not used sleep medication during the previous month.

**Table 2:** Distribution of quality of sleep

Variable items	Number	Percent
Sleep duration (h)		
0: ≥7	197	69.6
1: 6 ~ 6.9	66	23.3
2: 5 ~ 5.9	15	5.3
3: ≤4.9	5	1.8
Sleep disturbance (scores)		
0: 0	19	6.7
1: 1 ~ 9	246	86.9
2: 10 ~ 18	17	6.0
3: 19 ~ 27	1	0.4
Sleep latency (min)		
0: ≤15	142	50.2
1: 16 ~ 30	65	23.0
2: 31 ~ 60	46	16.3
3: ≥60	30	10.6
Daytime dysfunction		
0: Very good	213	75.3
1: Fairly good	49	17.3
2: Fairly bad	19	6.7
3: Very bad	2	0.7
Sleep efficiency (%)		
0: ≥85	248	87.6
1: 75 ~ 84	23	8.1
2: 65 ~ 74	7	2.5
3: ≤64	5	1.8
Subjective sleep quality		
0: Very good	32	11.3
1: Fairly good	162	57.2
2: Fairly bad	63	22.3
3: Very bad	26	9.2
Use of sleep medication		
0: Not during past month	237	83.7
1: Less than once a week	10	3.5
2: Once or twice a week	7	2.5
3: Three or more times a week	29	10.2
Global sleep quality		
Good quality (≤5)	204	72.1
Poor quality (> 5)	79	27.9

Results of the distribution of health-related fitness between good and bad sleepers using the Mann-Whitney U-test are presented in Table 3. Participants with bad sleepers reported significantly higher depression symptoms, chronic disease numbers, and up-and-go times than participants with a good quality of sleep. In terms of self-rated health, participants with a poor quality of sleep reported lower health conditions. Other anthropometric indices and physical fitness parameters did not significantly differ between participants with good and those with poor quality of sleep.

**Table 3:** Distribution of health-related fitness between good and bad sleepers Using Mann-Whitney U test (*N* = 283)

Variables	Good Sleepers (*n* = 207)	Bad Sleepers (*n* = 79)	z/p
	median (IQR)	median (IRQ)	
Age	58.00 (54.00–63.00)	57.00 (53.00–63.00)	−0.585/0.558
**Depression**	**0 (0–2)**	**4 (2–7)**	**−7.190/0.000**
**Chronic disease numbers**	**1 (0–1)**	**1 (0–2)**	**−2.125/0.034**
**Self-rate health**	**3 (3–4)**	**3 (2–3)**	**−5.399/0.000**
Anthropometric index			
BMI (kg/m2)	23.50 (21.50–25.80)	22.90 (20.85–25.45)	−1.278/0.201
FFMI	16.30 (15.40–17.41)	16.42 (15.50–17.33)	−0.050/0.960
Mid-arm circumference (cm)	28.90 (27.23–31.00)	28.60 (26.80–30.50)	− 1.239/0.215
Waist / hip ratio	0.85 (0.80–0.90)	0.84 (0.79–0.89)	−0.206/0.837
Physical Fitness			
Sit-and-reach (cm)	10.10 (1.60–16.50)	7.10 (0.00–13.83)	−1.351/0.177
One leg stance (sec)	60.00 (55.00–60.00)	60.00 (49.75–60.00)	−1.860/0.853
Two-min step (times)	113.00 (99.00–124.00)	107.5 (89.0–118.75)	−1.782/0.075
Chair stand in 30s (times)	16.00 (13.00–20.00)	15.00 (12.00–19.00)	−1.593/0.111
Grip muscle strength (kg)	24.10 (20.20–30.10)	23.30 (17.70–28.40)	−1.682/0.093
**Up and go (sec)**	**6.00 (5.00–6.00)**	**7.00 (6.00–7.00)**	**−2.324/0.020**
Back muscle strength (kg)	48.00 (31.50–64.50)	44.25 (30.38–59.00)	1.388/0.165

*BMI* Body mass index, *FFMI* Free Fat Mass Index, *IRQ* Interquartile range

Table 4 shows results of Spearman’s correlations between the PSQI and health-related fitness. In a basic condition, depression symptoms (*r* = 0.491, *p* = 0.000), and chronic disease numbers (*r* = 0.200, *p* = 0.001) were significantly positively correlated with the PSQI, and self-rated health (*r* = − 0.391, *p* = 0.000) was significantly negatively correlated with the PSQI. As to health-related fitness, sit-and-reach (*r* = − 0.131, *p* = 0.028), 2-min steps (*r* = − 0.133, *p* = 0.026), and up-and-go (*r* = − 0.169, *p* = 0.004) were significantly negatively correlated with the PSQI. Other health-related fitness factors were not significantly correlated with the PSQI (all *p* > 0.05).

**Table 4:** Spearman correlation between the Pittsburgh Sleep Quality Index (PSQI) and health-related fitness

	PSQI	
	r	*p*
Basic condition		
Age	.004	0.952
**Depression**	**.491**	**0.000**
**Self-rate health**	**−.391**	**0.000**
**Chronic disease (Numbers)**	**0.200**	**0.001**
Anthropometric index		
BMI	−.076	0.204
FFMI	.001	0.987
Waist / hip ratio	.011	0.857
Physical Fitness		
**Sit-and reach**	**−.131**	**0.028**
One-leg stand	−.080	0.179
**Two-min steps**	**−.133**	**0.026**
Chair stand in 30s	−.158	0.080
Grip muscle strength	−.105	0.195
**Up-and-go**	**−.169**	**0.004**
Back muscle strength	−.100	0.096

Results of the logistic regression analysis are presented in Tables 5 and 6 with each of the seven components of the PSQI and the global score as dependent variables. Covariates of age, sex, alcohol use, depression, number of chronic diseases, self-rated health, arthritis, and the four physical fitness items were used as independent variables. Results were as follows. Only depression was associated with a short sleep duration (OR = 1.172, *p* < 0.05); depression (OR = 1.225, *p* < 0.001) and arthritis (OR = 5.515, *p* < 0.05) were associated with sleep disturbances. Depression symptoms (OR = 1.096, *p* < 0.05), chronic disease numbers (OR = 0.604, *p* < 0.0), self-rated health (OR = 0.885, *p* < 0.01), and 2-min steps (OR = 0.985, *p* < 0.05) were associated with a longer sleep latency, and only depression (OR = 1.143, *p* < 0.01) was associated with daytime dysfunction. No other physical fitness items were associated with poor sleep efficiency; depression (OR = 1.260, *p* < 0.001) and self-rated health (OR = 0.856 and *p* < 0.001) were associated with poor subjective sleep quality; depression (OR = 1.148, *p* < 0.01) and self-rated health (OR = 0.869, *p* < 0.01) were associated with use of sleep medication; and depressive symptoms (OR = 1.220, *p* < 0.001), chronic disease numbers (OR = 0.524, *p* < 0.05), self-rated health (OR = 0.865, *p* < 0.001), and arthritis (OR = 4.562, *p* < 0.01) were associated with poor sleep quality. Among the controlling variables, sex, alcohol use, heart disease, chair stand in 30 s, up-and-go, and constant were not associated with any component of the PQSI.

**Table 5:** Logistic regression analysis of poor sleep quality with the global score and with seven components of the Pittsburgh Sleep Quality Index (PSQI) (*N* = 283)

Variable	Odds ratio							
	Poor sleep quality	Short sleep duration	Sleep disturbance	Longer sleep latency	Daytime dysfunction	Poor sleep efficiency	Poor subjective sleep quality	Use of sleep medication
Age	1.005	1.010	0.925	0.974	1.010	1.008	0.986	1.036
Sex	1.529	4.753	3.910	0.836	1.027	2.465	1.238	0.737
Alcohol consumption	0.573	0.876	1.015	0.790	0.826	1.747	0.648	0.405
CES-D score	1.220^***^	1.172^*^	1.225^***^	1.096^*^	1.143^*^	1.003	1.260^***^	1.148^**^
Chronic disease numbers	0.524*	0.404	0.8722	0.604*	0.629	0.830	0.650*	0.962
Self-rated health	0.865^***^	0.948	0.972	0.885^**^	0.960	0.926	0.856^***^	0.869^**^
Arthritis	4.562^**^	4.180	5.815^**^	2.38	2.126	2.922	2.959	1.622
Heart disease	2.296	NA	0.869	1.430	1.665	NA	1.046	2.298
Sit-and-reach	0.991	0.956	0.962	0.994	0.980	0.985	1.004	0.994
Two-min step	1.000	0.984	0.981	0.985^*^	1.003	0.998	0.993	0.993
Up-and-go	1.109	1.130	1.384	1.044	1.109	1.063	1.098	0.994

* *p* < 0.05, ** *p* < 0.01, *** *p* < 0.001

*CES-D* Center for Epidemiological Studies Depression Scale, *NA* there are no variability in the data for short sleep duration and poor sleep efficiency column

**Table 6:** Logistic regression analysis of poor sleep quality with the global score and with seven components of Pittsburgh Sleep Quality Index (PQSI) (*N* = 283)

Variable	95% Confidence interval							
	Poor sleep quality	Short sleep duration	Sleep disturbance	Longer sleep latency	Daytime dysfunction	Poor sleep efficiency	Poor subjective sleep quality	Use of sleep medication
Age	(0.960, 1.052)	(0.936, 1.090)	(0.846, 1.011)	(0.931, 1.018)	(0.941, 1.083)	(0.937, 1.084)	(0.943, 1.032)	(0.979, 1.095)
Sex	(0.625, 3.742)	(0.494, 45.703)	(0.512, 29.751)	(0.383, 1.825)	(0.283, 3.725)	(0.487, 12.475)	(0.528, 2.902)	(0.283, 1.923)
Alcohol consumption	(0.234, 1.404)	(0.173, 4.427)	(0.193, 5.338)	(0.356, 1.751)	(0.212, 3.222)	(0.503, 6.072)	(0.281, 1.497)	(0.114, 1.435)
CES-D score	(1.116, 1.333)	(1.043, 1.318)	(1.091, 1.376)	(1.015, 1.183)	(1.034, 1.262)	(0.884, 1.137)	(1.145, 1.387)	(1.051, 1.235)
Chronic disease numbers	(0.789, 6.684)	(0.165, 0.986)	(0.389, 1.951)	(0.382, 0.954)	(0.306, 1.294)	(0.401, 1.719)	(0.403, 1.049)	(0.568, 1.630)
Self-rated health	(0.792, 0.944)	(0.822, 1.093)	(0.833, 1.135)	(0.815, 0.961)	(0.838, 1.100)	(0.808, 1.063)	(0.785, 0.934)	(0.784, 0.963)
Arthritis	(1.513, 13.753)	(0.503, 34.773)	(1.115, 30.324)	(0.701, 5.926)	(0.410, 11.408)	(0.556, 15.351)	(0.995, 8.798)	(0.994, 5.329)
Heart disease	(0.315, 0.873)	NA	(0.148, 5.087)	(0.511, 3.999)	(0.339, 8.183)	NA	(0.358, 3.053)	(0.776, 6.808)
Sit-and-reach	(0.963, 1.020)	(0.914, 0.999)	(0.916, 1.011)	(0.967, 1.023)	(0.938, 1.024)	0.941, 1.031)	(0.975, 1.033)	(0.961, 1.028)
Two-min step	(0.986, 1.015)	(0.960, 1.009)	(0.954, 1.010)	(0.972, 0.999)	(0.980, 1.028)	(0.975, 1.021)	(0.979, 1.007)	(0.975, 1.011)
Up-and-go	(0.890,1.415)	(0.767, 1.666)	(0.902, 2.123)	(0.826, 1.319)	(0.761, 1.616)	(0.726, 1.554)	(0.865, 1.393)	(0.752, 1.315)

*NA* there are no variability in the data for short sleep duration and poor sleep efficiency column

## Discussion

Our study have two major findings: (1) depression symptoms, the number of chronic diseases, self-rated health, and arthritis were significantly associated with bad sleepers; and (2) the 2-min step test was associated with longer sleep latency.

These results confirmed the correlation of health-related physical fitness with sleep quality in the middle-aged and elderly, and among the health-related physical fitness items measured, only the 2-min step test was associated with longer sleep latency. Our study revealed that 27.9% of participants reported bad sleepers. We discovered that 10.2% of study participants frequently used sleep medication to help them fall asleep, and 6.0% reported having significant depressive symptoms (CES-D ≥ 10). The components of sleep quality evaluated in this study were diverse, and sit-and reach, 2-min step test, and up-and-go physical fitness items were significantly correlated with components of sleep quality; but only the 2-min step test was associated with longer sleep latency after controlling for sex, age, current number of chronic diseases, self-rated health status, and depressive symptoms confounding factors. However, depressive symptoms, the number of chronic diseases, self-rated health status, and arthritis were main risk factors influencing bad sleepers among the middle-aged and elderly.

We found that depressive symptom caused a short sleep duration. Gerber et al. [22] reported that low fitness levels and a perceived lack of physical activity were associated with longer sleep onset latency. We similarly found better 2-min step endurance was associated with a lower risk of longer sleep latency. In our questionnaire, daytime functions, including driving, eating meals, and engaging in social activities, were less affected by depression. Lee and Lin [16] reported greater sit-and-reach distances in young women with favorable sleep quality, and we found that fewer depressive symptoms and chronic disease numbers, and better self-rated health were associated with subjective good sleepers. Furthermore, the self-rated health status also had major influences on sleep latency, subjective sleep quality, and global sleep quality.

In this study, the logistic regression analysis disclosed associations of sleep disturbances and poor sleep quality with arthritis. Patients with RA suffer from a variety of symptoms such as joint pain, fatigue, stiffness, sleep disturbances, and functional disability [7]. High prevalence of abnormal sleep quality were observed in both RA and osteoarthritis patient populations. The most common abnormality was sleep fragmentation, with an increased sleep disturbance score [23]. Only 18.5% of RA patients reported good sleepers, with depression and risk of sleep apnea being independently associated with sleep impairment [24].

Older adults with a long sleep duration had weaker hand grip strength irrespective of muscle mass [25]. More muscle power corresponded to a lower probability of using sleep medication. Upper-arm muscle strength was a factor related to sleep medication use [24]. We found that sleep medication use was associated with self-rated health and depressive symptoms. Approximately 90% of patients with depression reported poor sleep quality [26], for which depressive symptoms may have a greater influence than physical fitness.

This study has several limitations, including a small sample size, an unbalanced number of men and women recruited, sleep measurement based on subjective descriptions, and the lack of a control group. Furthermore, many factors, such as those pertaining to genetics and the environment, were not considered in our study and may have influential effects on sleep quality [27].

## Conclusions

Beyond sleep duration, the quality of this time is important to consider. Middle-aged and older adults are more likely a result of declining sleep quality and duration associated with aging. Although Sleep quality is typically harder to measure because it can be subjective, with regard to cognition and health. Measurement of health-related physical fitness, that is, fitness related to disease prevention and health promotion. This study revealed that depressive symptoms, the number of chronic diseases, self-rated health status, and arthritis were the main risk factors that influenced global sleep quality among the middle-aged and elderly who participated in this study; and the 2-min step test for physical fitness was associated with longer sleep latency. The 2-min step test requires no expensive equipment and only a few square meters of space, has been used internationally with both healthy and diseased individuals, further research is required regarding the validity, reliability, and responsiveness of the test.

## Data Availability

The datasets are not publicly available due to ethical reasons, but are available from the corresponding author on reasonable request.

## Abbreviations

BMI: Body-mass index
CES-D: Center for epidemiologic studies depression scale
FFMI: Free fat mass index
IPAQ: International physical activity questionnaire
PSQI: Pittsburgh sleep quality index
